# Decision of the Hon. George Parker, Judge of the Supreme Court, Regarding the Legality of the Charter of the College of Physicians and Surgeons of Buffalo

**Published:** 1881-09

**Authors:** 


### BUFFALO

### Medical and Surgical Journal.

Vol. XXI. SEPTEMBER, 1881. No. 2.

### Original Communications.

### Decision of the Hon. George Barker, Judge of the Supreme Court, Regarding the Legality of the Charter of the College of Physicians and Surgeons of Buffalo.

In the suit brought to test the validity of the charter under which the College of Physicians and Surgeons of Buffalo is conducted, Hon. George Barker of the Supreme Court, before whom the matter was argued at Special Term, has overruled the demurrer of the defendants and gives leave to answer the complaint in twenty days, on payment of costs; otherwise absolute judgment will be awarded to the plaintiff. The Judge hands down an elaborate opinion, of which the following is the full text:

Supreme Court.—The People of the State of New York *vs.* George W. Cothran, Zebulon Ferris, Henry Childs, Jerome F. Fargo, Stephen O. Barnum, Alfred B. Southwick, Samuel W. Wetmore, Garrett C. Daboll, Thomas P. Sears, George W. Cutter and Samuel N. Brayton.

Erie County Special Term.—June, 1881.

Hamilton Ward, Attorney-General, and Lewis & Rice for the People.

Tracy C. Becker and George W. Clinton for the defendants.

BARKER, J.—The Attorney-General, upon his own information, prosecutes this action against the defendants, charging them with acts of usurpation, in seeking to create and maintain a body corporate, without due authorization from the legislative authority of the State. The Attorney-General, in prosecuting the action, stands upon the provisions of section 948 of the Code of Civil Procedure, which provide that "the Attorney-General may maintain an action, upon his own information, against one or more persons who act as a corporation within the State without being duly incorporated, or exercise within the State any corporate rights, privileges or franchises not granted to them by the law of the State."

In March, 1879, the defendants filed a certificate which in form is a compliance with chapter 319 of the laws of 1848, entitled "An act for the incorporation of benevolent, charitable, scientific, and missionary societies," was the same amended at that time. They were named in the certificate as the trustees of the corporation, and have ever since acted in that capacity, and as the officers and agents of the corporation intended to be created.

The name of the society as mentioned in the articles of association is "The Buffalo College of Rational Medicine." The objects and purposes of the society, as mentioned therein, are to create, establish, maintain and conduct in the city of Buffalo a college for instruction in medicine, surgery and dentistry, and in all science connected therewith, and ancillary thereto, and when deemed expedient to establish, maintain and conduct in connection therewith a free medical dispensary.

This certificate was endorsed and approved by one of the Justices of the Supreme Court.

It is alleged in the complaint "that on or about the 20th day of March, 1879, the defendants associated themselves together under the name of 'The Buffalo College of Rational Medicine,' and gave out and pretended to be incorporated as a medical college, and assumed and did act as a corporation and performed pretended corporate acts; that they established a pretended medical college in the city of Buffalo, and pretended to give instruction in the art of medicine and surgery, for which they demanded and received compensation, and issued and granted diplomas to graduates, and pretended to do all other acts pertaining to and ordinarily done by incorporated medical colleges."

The complaint also contains an averment that the defendants did not claim to have any other authority for maintaining a corporation of the character mentioned, except such as is contained in the act of 1848, and the several acts amendatory thereof.

To this complaint the defendants have interposed a general demurrer, claiming that the complaint does not state facts sufficient to constitute a cause of action.

The legal proposition presented by this demurrer is whether a corporation with the powers, functions and attributes of a medical college proper can be created and brought into existence under the provisions of the act already mentioned, and as such body corporate confer the degrees of learning usually bestowed by incorporated colleges, and in testimony of such grant to give suitable diplomas, which shall entitle the possessor to all the immunities and privileges which the laws and customs of the country confer on such persons.

Intermediate the passage of the original act and the time of filing the certificate of organization by the defendants, the first section of the act of 1848 was several times amended, changing and enlarging the powers and purposes of such corporations as should be organized under the act. The last amendment was in 1872, chapter 649, when the text of the first section was rewritten, and as amended reads as follows: "That five or more persons may associate themselves together for benevolent, charitable, literary, scientific, missionary or mission and other Sunday-school purposes, or for the purpose of mutual improvement in religious knowledge, or the furtherance of religious opinion, or for any two or more of such objects combined."

Societies organized under this statute possess the general powers conferred by, and are made subject to, the provisions and restrictions of title 3 of the 18th chapter of the third part of the Revised Statutes.

In considering the legal proposition, I shall only discuss the merits of the question, disregarding all mere technical criticisms which have been made as to the sufficiency of the complaint, as the learned counsel for the respective parties upon the argument expressed a desire to reach and have adjudicated upon the trial of this demurrer the vital question in issue, so important to the defendants as individuals as well as to the public at large, whether a corporation with the powers claimed by the defendants has been lawfully brought into existence.

After an examination of the provisions of the general act in question, together with concurrent legislation authorizing the creation of literary and medical colleges, I am well satisfied that the legislature did not intend by the provisions of the act of 1848 to allow the formation of corporations with the powers and functions possessing the rank and dignity of literary or medical colleges proper; that corporations formed under the act of 1848 were intended to serve a less important and different purpose, and for that reason they have been exempted from many of the restraints, obligations and duties imposed upon institutions of the other and higher grades.

In considering the question, and in examining the acts referred, it will be useful and to some extent necessary to refer to the history of legislation in this State in regulating the practice of physic and surgery, and the purpose and policy of the laws relating thereto.

The science of medicine and surgery has been to some extent fostered by the State from its earliest history, and institutions of learning have been organized to teach and instruct persons who intend to practice the profession of medicine as a vocation in life that the public may enjoy the services of learned and skillful physicians. Prohibitory statutes were also at the same time passed declaring that no person should practice physic or surgery without having a license therefor, emanating from some institution or board of examination well qualified to determine who were suitable persons to engage in the important and delicate duty of visiting individuals and families in the professional capacity of a physician. The unauthorized practice of physic and surgery was made a misdemeanor, punishable by fine or imprisonment, or both.

Later in the history of legislation upon this subject, and I believe in the year 1844, an act was passed which swept away all criminal and penal laws against the unlicensed practice of physic and surgery and abolished all laws which prohibited any person from receiving a compensation for services as a physician or surgeon, whether licensed or not. Notwithstanding the radical change the statutes continued to provide for a system of granting licenses and issuing diplomas as heretofore had been the custom, and but few men were bold enough to offer themselves to the public as medical practitioners and surgeons who did not have one or the other of the certificates of fitness granted to them by public authorities.

In 1880, chapter 513, the policy of free license in the practice of medicine was completely terminated, and a return to the former system was inaugurated, and by the terms of this last mentioned act it is provided that no person shall practice physic or surgery within the State unless he is twenty-one years of age, and has been authorized in the manner required by law regulating the granting of licenses and diplomas. Any person violating these requirements and engaging in the practice of medicine without license or a diploma is made guilty of a misdemeanor, and may be punished by fine or imprisonment, or both. It is also provided by the fifth section of the act of 1880, that the degree of Doctor of Medicine, lawfully conferred by any incorporated medical college or university, shall be a license to practice physic and surgery. The only other authorized mode of securing a license authorizing the practice of medicine, is by complying with the requirements of the law of 1872, chapter 746.

By this act it is provided that the Regents of the University shall appoint one or more boards of examiners in medicine, each board to consist of not less than seven persons, who shall have been licensed to practice physic and surgery in this State. Such examiners shall faithfully examine all candidates referred to them for that purpose by the Chancellor of the University, and furnish him a detailed report in writing of all questions and answers of each examination, together with a separate written opinion of each examiner as to the requirements and merits of the candidate in each case. "Such examination shall be in anatomy, physiology, materia medica, pathology, histology, clinical medicine, chemistry, surgery, midwifery and in therapeutics, according to each of the systems of practice represented by the several medical societies of this State." No person is entitled to be so examined before such board unless he is over twenty-one years of age, of good moral character, and is able to produce proofs satisfactory to the Chancellor of the University that he is of competent knowledge in all the branches of learning taught in the common schools of this State and of the Latin language, and that he diligently studied medicine, not less than three years, under the direction of one or more physicians duly qualified to practice medicine, or has himself been duly licensed on examination by some medical society or college legally empowered to issue licenses or degrees in medicine, and upon paying to the treasurer of the university the fees mentioned in the fith section of the act.

By the general law of the State authorizing the formation of medical colleges (chapter 184 of the Laws of 1853), the trustees of such medical colleges are given power to grant and confer the degree of Doctor in Medicine upon the recommendation of the board of professors of such college, and at least three curators of the medical profession appointed by said trustees, but no person shall receive a diploma conferring such degree unless he be of good moral character, and of the age of twenty-one years, and shall have received a good English education, and shall have pursued the study of medicine and the science connected therewith for at least three years after the age of sixteen, and have received instruction from some physician and surgeon fully qualified to practice his profession until he is qualified to enter a medical college and shall also after that age have attended two complete courses of lectures, delivered in some incorporated medical college.

This brief statement of the nature and character of the past and present laws on the subject of licensing persons to practice medicine, and of the attainment and qualifications which are required of persons who may receive such permit, and the pains and penalties that follow from a non-observance of these particular requirements, will present to every intelligent mind at once the importance of the subject under consideration, that it is a question of both public and private concern.

The history and course of legislation in passing general laws for the formation of corporations of various kinds and character which are required, and are necessary in a populous, commercial, trading, manufacturing and intelligent people, indicates very clearly, if it does not altogether demonstrate, that it was not the intention of the Legislature by the provisions of the act of 1848 to authorize the formation of a medical college as such institutions are known to the law and customs of the State.

Since the constitution of 1846 it is the policy of the State that all corporations of every grade and character, except municipal, should be organized under general laws, and that special legislation was not to be resorted to for such purposes, although not in terms prohibited by the constitution.

In pursuance of the spirit of the fundamental law of the State, many general laws have been passed authorizing the formation of corporations of the various classes, such as banking business and trading, commercial and manufacturing, with such powers and functions, obligations and restrictions as in the wisdom of the Legislature should be given and imposed upon such corporations.

By the laws of 1853, chapter 184, entitled "An act relative to the incorporation of colleges and academies," the regents of the university by general rules and regulations were required to prescribe the requisites and conditions of the incorporation of colleges, universities and academies, or other institutions of learning, pursuant to the power vested in said regents by an act relative to the university, passed April 5, 1813, as amended by the Revised Statutes of this State.

In this same act special and particular conditions were exacted on organizing medical colleges, and such institutions were required to have an endowment of fifty thousand dollars before a charter would be given them. Special provision was made as to the amount of property such corporations might take and hold, and limiting the uses and purposes to which the funds and moneys of the corporation could be used, and making them subject to the general provision of the Revised Statutes, so far as the same was applicable to regulating the practice of physic and surgery within this State, and giving them the power to confer a degree of medicine, and to issue diplomas to the class of persons possessing the qualifications which have been hereinbefore mentioned.

By a general provision of the Revised Statutes, there is conferred upon every college, besides the general powers and privileges given it by the law of its incorporation, the right and function to grant such literary honors as are usually granted by any university, or college or seminary of learning within the United States, and in testimony thereof to give suitable diplomas, under seal and the signature of such officers of the college as they shall deem expedient, and every diploma granted by such college shall entitle the possessor to all the immunities, which by usage or statute are allowed to possessors of a similar diploma granted by any university, college, or seminary of learning in the United States.

Reading the act of 1848 and the amendments thereto, in connection with these other general laws, it is obvious that the intention of the legislature was to authorize the formation of corporations for purposes distinct from that of literary or medical colleges proper.

By giving to the act of 1848 an interpretation limiting the powers of corporations created under it, so as not to embrace a medical college proper with the powers, functions and attributes bestowed upon such institutions, the system of general laws relating to the creation of colleges is made harmonious, each act consistent with the other, and gives to the legislation of the State a rational and intelligent meaning. To hold to the contrary, that medical and literary colleges to be known as such can be organized under the act of 1848 as well as under the act of 1853, would make the system of laws on the subject inharmonious, confused and misleading. I am convinced that it was not the intention of the Legislature to suffer and permit medical colleges to be organized and maintained, possessing the powers which are bestowed upon such institutions, without at the same time placing them under any other obligation and restraint than is imposed by the act of 1848.

If the position of the defendant is maintained, then the Medical College of Rational Medicine at Buffalo, of which the defendants are trustees and officers, can graduate students, confer degrees, issue diplomas, without the graduate having any other qualifications than such as may be imposed by the defendants themselves. I think that all persons who may give to this subject a thorough examination, study the history of the State relating to such matters, and examine the laws which have been referred to, will readily reach the conclusion that it would not be wise and safe to bestow upon individuals who may voluntarily associate themselves together under the act of 1848 the right of establishing the degree of qualifications which graduates of medical colleges shall possess, and without being required to inquire into their moral character and fitness to practice medicine.

In the best interests of the public and families it is required that all graduates from medical colleges organized under the law of 1853, or applicants who may receive a license to practice medicine from the Board of Regents should possess the qualifications which are in the acts themselves especially enumerated.

Within the last fifty years there have been created many literary and medical colleges by special enactment; and as bearing up the proposition that a corporation organized under the act of 1848 has no power to organize a college proper, either literary or medical, it will be found instructive to consider the provisions of these several special enactments, as indicating the policy of the State to be as already stated; and it will be found that in instances where the college so incoporated is of a literary character it has been clothed with the power to grant diplomas, and when a medical college proper has been organized, the power is given to grant a diploma conferring the degree of Doctor of Medicine, but in every instance requiring as a prerequisite that the possessor shall have pursued a course of medical studies such as are prescribed in the several acts of incorporation. I quote from the charter of the University of Buffalo, which institution has the power of granting diplomas conferring the degree of Doctor of Medicine. Most of the special charters contain side provisions: "No person shall receive such diploma unless he shall have pursued the study of medical science for at least three years after he attains the age of sixteen years with some physician and surgeon duly authorized by law to practice his profession, and shall also after that age have attended two complete courses of all the lectures delivered in some incorporated medical college, the last of which courses shall have been delivered by the medical faculty of such university."

Reference is also made to the charters of the New York Medical Society, chapter 206 of the laws of 1850; the New York College of Dental Surgery, the laws of 1852, chapter 261; the charter of the People's College, laws of 1853, page 193; the Metropolitan Medical College of the City of New York, laws of 1854, chapter 192; the New York College of Veterinary Surgeons, chapter 267 of the laws of 1859; the Excelsior Medical College in the City of New York, chapter 160 of the laws of 1858; the American College of Medical Science, chapter 85 of the laws of 1858.

The defendants have cited chapter 51 of the laws of 1870, which is an amendment of the act of 1848, and argue that under the provisions of the first section of this act authorization is given to incorporate any society for the purpose of establishing and maintaining any educational institution, and that by its terms power is given in terms broad enough to authorize the organization of a college proper.

I do not concur in the interpretation which the learned counsel for the defendants have placed upon this first section, but am of the opinion that the true interpretation is to limit this provision to religious societies entirely.

I am also of the opinion that this section is repealed by chapter 647 of the laws of 1872, hereinbefore referred to, when the first section of the act of 1848 was re-written and re-enacted providing for the formation of societies of the character mentioned in the act of 1870, and to ascertain the powers which corporations possess organized since 1872. Reference must be had to the first section of the act as last amended, and be determined thereby. The act of 1870 was not amended to stand independent of the act of 1848 in all its provisions, for by the title of the act it is declared to be an amendment to the act passed April 12th, 1848.

It is further argued and maintained by the defendants' counsel that the same act of 1870 gives the legislative interpretation as to the right to organize colleges and universities under the act of 1848. It must be held in view of the previous and concurrent legislation which has been hereinbefore alluded to, that the words "university" and "college," as used in this section, are used in a limited and modified sense, and that it was not the purpose by the use of these terms to signify the intention that a university or college proper could be created in the easy and unceremonious way in which a charitable and benevolent society could be organized under the general act of 1848.

The act of 1870 can have full significance and effect given to all its provisions without yielding to such liberal interpretation.

Beyond all doubt a corporation may be organized under the act of 1848, for the purpose and object stated in the certificate made and filed by the defendants, that is to say, to establish, maintain and conduct a society for instruction in medicine, surgery and dentistry, and in all sciences connected therewith and and ancillary thereto, and to maintain and conduct a free medical dispensary. Such purposes are in their character benevolent, charitable and scientific, as well as useful and beneficial to the community. Such an institution, properely organized and conducted, would be a school with praiseworthy and commendable objects and purposes.

But the defendants, by their demurrer, have admitted that their object and purpose in seeking to organize this corporation, is to create and maintain a medical college as such institutions are known to the laws of the State, and to confer honorary degrees and grant diplomas to its graduates.

The demurrer is overruled, with leave to answer on payment of costs within twenty days; if costs not paid and answer made within this time, then judgment absolute for the plaintiff.

